# Editorial: What Determines Social Behavior? Investigating the Role of Emotions, Self-Centered Motives, and Social Norms

**DOI:** 10.3389/fnhum.2016.00342

**Published:** 2016-07-07

**Authors:** Corrado Corradi-Dell'Acqua, Leonie Koban, Susanne Leiberg, Patrik Vuilleumier

**Affiliations:** ^1^Swiss Centre for Affective Sciences, University of GenevaGeneva, Switzerland; ^2^Laboratory for Neurology and Imaging of Cognition, Department of Neurosciences and Clinic of Neurology, University Medical CenterGeneva, Switzerland; ^3^Département de Psychologie, Faculté de Psychologie et des Sciences de l'Éducation, Université de GenèveGeneva, Switzerland; ^4^Department of Psychology and Neuroscience, University of ColoradoBoulder, CO, USA; ^5^Department of Economics, University of ZürichZürich, Switzerland

**Keywords:** social behavior, ultimatum game, emotions, decision making, contextual appraisal, medial prefrontal cortex (mPFC), autism spectrum disorders (ASD), oxytocin receptor gene

In the last decade, a growing research effort in behavioral sciences, especially psychology and neuroscience, has been invested in the study of the cognitive, biological, and evolutionary foundations of social behavior. Differently from the case of sociology, which studies social behavior also at the *group* level in terms of organizations and structures, psychology and neuroscience often define “social” as a feature of the *individual* brain that allows an efficient interaction with conspecifics, and thus constitutes a possible evolutionary advantage (Matusall). In this view, an extremely wide range of mental and neural processes can be classified as “social,” from the coding of relevant sensory stimuli about conspecifics (facial expressions, gestures, vocalizations, etc.), to the selection and planning of behavioral responses in complex interpersonal settings (economic transactions, negotiations, etc.). Despite such heterogeneity, there is a converging interest in the scientific community toward the identification of neural and psychological mechanisms that underlie all the many facets of social behavior, and their comparison across species and cultures.

This Research Topic was initiated by researchers from the Swiss National Center of Competence in Research “Affective Sciences—Emotions in Individual Behaviour and Social Processes,” a multidisciplinary institution devoted to the study of affect-related processes across various disciplines (from psychology and neuroscience through to history, philosophy, art, and economy). In keeping with this spirit, this Research Topic comprehends 38 contributions from an interdisciplinary community each addressing specific psychological and neural phenomena that can be defined as “social.” In particular, we collected both theoretical and empirical contributions, concerning animals, human individuals (neurotypical adults and children, but also individuals with neurological, psychiatric and developmental disorders) as well as human groups, engaged in either laboratory-controlled settings or real-life situations. Although the theoretical models and the applied research techniques (psychophysical, physiological, neuroimaging, genetic) are very diverse, they converge with a global framework suggesting that the determinants of social behavior can be described across two independent dimensions: (1) a *personal-to-environmental* dimension, and (2) a *transient-to-stable* dimension. These contributions thus represent an important cornerstone for building an interdisciplinary and comprehensive model of how individuals deal with the complexity of their social environment.

## Personal-to-environmental dimension

For the purpose of this editorial, we can schematically describe social interactions as cases in which an individual is engaged in a given social environment. Importantly, the individual and the environment exert reciprocal influence on one another, as individual changes could cause, and be caused by, changes in the outside world. Within this context, we can define a behavior of interest any change of the individual's state over time (overt response, brain modulation, etc.), which in turn can be related to two main explanatory variables: a representation of the current state of the individual (to know how a person will change one needs to know how this person is) and a representation of the current state of the environment (to know how a person will change one needs to know what surrounds him/her). Thus, the *personal-to-environmental* dimension distinguishes between those determinants of social behavior that are attributable to idiosyncratic features of the individual from those that are related to specificities of the environment with which the individual is interacting. Such simplified model fits well our Research Topic, as the various contributions highlight the role of many factors that, despite their diversity, can be readily classified as personal or environmental.

Among the personal factors, the role played by genetic polymorphisms is well-described in the present Research Topic through the use of knock-out mice and endophenotype approaches in humans. In all these cases, the implicated genes are known to affect major functions of hormonal and neurotransmitter systems within brain networks important for social cognition. For instance, mice lacking the β2 subunit of neuronal nicotinic receptors of acetylcholine exhibit impaired behavior (relative to wild type mice) when competing with conspecifics for rewards (Chabout et al.) Furthermore, following a rich body of literature documenting how intranasal administration of oxytocin affects human social behavior (see Ebner et al; Haas et al.; Järvinen and Bellugi, as reviews), several contributions address the role played by the oxytocin gene receptor (OXTR). Taking a developmental perspective, Ebner et al. show how OXTR polymorphisms differently affect young and older adults' responses in medial prefrontal cortex (MPFC) to facial emotional expressions. Haas et al. suggest how OXTR polymorphisms might explain variations in individual cooperative behavior by affecting the structure and function of key brain areas for social behavior such the amygdala, the superior temporal sulcus, and the anterior cingulate cortex. It is possible that brain regions with high density of oxytocin receptors (such as the amygdala) affect social behavior through their regulatory role on the autonomic nervous system, an hypothesis put forward by Järvinen and Bellugi to account for social dysfunctional behavior in Williams Syndrome, in addition to more classic effects on cognition or learning. Finally, Hruschka and Henrich point out that genetic polymorphism might even explain some cultural differences, as suggested by the controversial evidence that collectivistic (as opposed to individualistic) societies might most frequently exhibit allelic variation of serotonin-transporter-linked polymorphic region (Chiao and Blizinsky, 2010; Eisenberg and Hayes, 2011).

A few studies also highlighted the role played in social behavior by individual *traits*: these are habitual patterns of behavior, thoughts and emotions that are relatively stable over time. Although of unclear etiology, inter-individual trait variability has been often used in the literature as a powerful factor that explains behavioral differences in the neurotypical population. This is the case of several studies from the present Research Topic, who report for instance that individual empathic traits can influence the decoding of emotional facial expressions Huelle et al., or monetary decisions on behalf of unknown people (O'Connell et al.). Furthermore, (Maresh et al.) find that the neural response to electrical shocks (and the degree to which this is affected by social proximity) is modulated by individual anxiety trait, a measure of idiosyncratic sensitivity to stressors. Finally, this Research Topic includes multiple studies on individuals exhibiting traits diagnostic of psychopathy, a developmental syndrome characterized by low levels of empathy, guilt, and remorse, but increased aggressive and antisocial behavior (Marsh). In particular, individuals with high psychopathic scores exhibit altered neural and behavioral responses in many experimental manipulations related to fear conditioning (Veit et al.), fear empathy (Marsh), or moral cognition (Tassy et al.). The case of psychopathy highlights the close tie between individual traits and the presence of disorders, which can be considered in some cases as extreme variants of normative behavioral patterns (Hare and Neumann, 2005; Walton et al., 2008). Consistently, several studies report atypical social behavior in individuals with psychiatric diagnoses or neurodevelopmental syndromes. For instance, individuals with schizophrenia and bipolar disorders show impairments in tasks involving the inference of others' thoughts and emotions (Caletti et al.). In a similar vein, individuals with Autism Spectrum Disorder or Asperger Syndrome display atypical behavior in several tasks (see Zalla and Sperduti, for review) ranging from visual processing of emotional facial expressions (Corradi-Dell'Acqua et al). to the inference of others' states, empathy, and moral cognition (Baez et al.).

Among environmental factors, several studies in the present Research Topic highlight the role played by social norms. These can be understood as representations of community's desires and expectations about end states that guide our evaluation of events and the selection of behavioral responses (see Brosch and Sander, for more details on norms and values). In particular, Hruschka and Henrich point out that socioeconomic rules (related to religion or market) can explain the degree to which populations are eager to exhibit in-group biases. Furthermore, Clément and Dukes discuss how one's interest toward events in the environment might be biased by their *normative significance*, i.e., by the degree to which these are relevant for social norms and for the self-concept in the community. Additional contributions suggest how people's behavior during situations involving division of goods can be understood prevalently in terms of fairness norms or equality heuristics, according to which people are eager to sanction unequal divisions even at their own expenses (Civai). For instance, Shaw and Olson show that children from 6 to 8 years of age will correct (or at least attempt to minimize) unequal distributions of tokens between two unknown kids. In adults, two articles suggest a major role of fairness heuristics in the well-known Ultimatum Game task (Civai; Guney and Newell): in both cases the authors argue that individuals (responders) refuse money which is freely offered to them when part of an unequal division, regardless of their ongoing emotional response (Civai) or of the alleged intentions of person (the proposer) who is making the offer (Guney and Newell).

## Stable-to-transient dimension

Most of the studies reviewed in the previous section describe factors that, despite their difference, can be classified as *stable*, i.e., they are held to exert a long-lasting effect on individual social behavior. These can be understood as general behavioral determinants, which transcend specific situations. Although important, stable determinants have only an approximate predictive power, as a large variability of individual social behavior can be explained in terms of *transient* factors related to the specificities of the interpersonal situation. For instance, as individual social behavior can be partly explained by idiosyncratic features of the individual, they can as well be affected by factors that temporally alter the individual's state and how he/she interacts with the social environment.

Several studies document that people's social behavior can be affected by manipulating their preexisting emotional state, for instance by showing them arousing stimuli, exposing them to stressful vs. rewarding conditions, or engaging them in emotion regulation strategies. As for the case of genetic polymorphisms, these preexisting emotional states can alter the mental and brain processes critical for individual social behavior, thus showing how affective and social functioning might rely on partially overlapping systems. For instance, Eskine presents compelling evidence that people's moral coding might be grounded in the same processes underlying gustatory disgust (see also Eskine et al., 2011, 2012). Likewise, in line with a rich body of literature showing how empathetic reactions to others' pain and disgust recruit similar neural structures as those involved in first-hand experiences of pain and disgust (Corradi-Dell'Acqua et al., 2011, 2016; Bernhardt and Singer, 2012, but see Krishnan et al., 2016), Marsh argues that dysfunctions in fear experience might lead to a reduced capacity to recognize fear in others (see also Adolphs et al., 1994).

Several contributions examine the role of preexisting emotional states in decision-making using behavioral economics paradigms. The theoretical framework underlying most of these studies posits that individual decisions result from the interaction of at least two different brain systems (Dual-System model—see Halali et al.): the cognitive/deliberate system (slow, controlled, cognitively-demanding, and instantiated mainly in prefrontal cortex) and the affective system (fast, automatic, cognitively non-demanding, and instantiated predominantly in limbic regions). As these two systems might promote conflicting courses of actions, transient emotional induction can be used as a mean to strengthen the affective contribution to a decision, as shown by Eimontaite et al. who find that inducing anger in people makes them less cooperative in social decision-making tasks such the Trust Game and the Prisoner Dilemma. Using a complementary approach, some studies engaged participants in emotion regulations strategies, by asking them to up- or down-regulate their emotional responses. Such regulation was found to have a significant impact on subsequent behavior (Grecucci et al.; van't Wout et al.) and brain responses (Grecucci et al.) in tasks such the Ultimatum and Dictator Game.

## Contextual and social appraisal

Accounts such as the Dual-System Model have been criticized for their dichotomous separation between cognition and emotion, which appears oversimplistic and not supported by empirical evidence (e.g., Moll et al., 2008; Shackman et al., 2011; Koban and Pourtois, 2014; Phelps et al., 2014). Alternative theoretical frameworks suggest instead that emotion is not a unitary construct opposed to cognition, and that distinct affective/motivational components may impact behavior in different (and in some cases opposite) ways (Moll et al., 2008; Phelps et al., 2014). In particular, appraisal theories of emotions (e.g., the Component Process Model by Scherer, 1984, 2009) propose that affective experience is critically determined by a series of cognitive evaluations (appraisal checks) of the environment in terms of events' novelty, valence, impact on one's goals, and how they can be dealt with. For instance, sadness is based on the awareness of the presence of a salient negative event (e.g., the occurrence of a terminal disease), undermining personal goals (it will end one's life), against which no course of action seems effective. The same event can instead induce an emotional response of higher arousal (such as anger or rage), if associated with the belief that a solution (a treatment) is available. In this perspective, the Component Process Model is not merely a theory of emotions, but can be seen as a comprehensive framework in which cognitive evaluation of the environment, affective reactions, and preparation of a behavioral response are integrated into a unique system.

For the purpose of this editorial, the appraisal checks proposed by the Component Process Model (Scherer, 1984, 2009) are good candidate processes for explaining how the social environment should not be considered as a stable construct exerting long-lasting effects on individual behavior, but also as the result of multiple contextual or transitory factors that, when combined together, make each inter-personal situation unique. In accord with this view, several contributions to this Research Topic suggest that individual affective and behavioral responses might be determined by evaluations of the social context, some of which correspond to the same appraisal checks described in the Component Process Model. For instance, Maresh et al. show that, in anxious individuals, neural responses to threatening electrical stimuli are modulated by whether participants are alone or close to a person that could be a stranger or a friend. Furthermore, Clark-Polner and Clark review how interpersonal behavior (e.g., reaction to others' emotions, providing and receiving social support) are affected by the context of the relationship. Similarly, Baez et al. suggest that the social proficiency of individuals with Asperger Syndrome could improve when the contextual information from social settings is made explicit. Finally, Alexopoulos et al. had participants playing as responders in a modified Ultimatum Game task, and find that the neural activity in MPFC to unfair offers is affected by whether they could retaliate against the proposer (which reflects a change in coping potential).

Due to the dynamic properties of interpersonal relationships and interactions, simple appraisal checks such the assessment of novelty, valence, coping potential, etc. are often not sufficient to tackle the complexities of social situations. Among the many contextual/transitory properties of the environment that need to be appraised, there is also the presence of other human beings, each with their own mental states and cognitive appraisals. Let's imagine, for instance, the case in which an individual is observing a friend, in the attempt to infer his/her emotional states. It is reasonable that, to do so, the individual might model the behavior of the observed friend in relation of the most likely determinants, including his/her contextual appraisal. In particular, the individual can assess if the friend is sad, by checking whether he/she believes to be terminally ill and that a treatment might not be available (see also Corradi-Dell'Acqua et al., 2014). This is an example of *social appraisal*, in which each individual represents contextual aspects of the social environment also in terms of how other bystanders evaluate the same environment from their point of view (see Manstead and Fischer, 2001; Clément and Dukes). Social appraisal refers to individuals' metacognitive abilities, and has close ties with concepts such as mentalizing, theory-of-mind, and perspective taking. Importantly, the role played by social appraisal has been highlighted in this Research Topic by articles focusing on impression formation (Kuzmanovic et al.), interpersonal relationships (Bombari et al.) and monetary transactions (Halali et al.; Tomasino et al.). In particular, the behavioral and neural responses of individuals (responders) to unfairness in the Ultimatum Game can be affected by whether the monetary transaction is framed by the proposer in terms of offer (“I give”) or acquisition (“I take”; Sarlo et al., 2013; Tomasino et al.) Furthermore, Halali et al. suggest that, when playing as proposers in the Ultimatum and Dictator Game tasks, participants most automatic choices are driven by considerations about whether the responder can retaliate against a potential unfair treatment.

Social appraisal can be differentiated from other kinds of contextual evaluations at the neural level. In particular, in line with existing models on the organization of MPFC (Lieberman, 2007; Forbes and Grafman, 2010; Corradi-Dell'Acqua et al., 2015), Bzdok et al. use meta-analytical evidence to propose a segregation between a dorsal portion, involved in top-down, controlled, metacognitive abilities, and a ventral portion involved in bottom-up, automatic evaluative-related processes. This segregation is also supported by Kang et al. who show how the dorsal MPFC is implicated in accurately estimating other people's preferences, whereas the ventral MPFC is recruited when using the Self as a proxy for the estimation. Furthermore, Grossmann reports that, already at the age of 5 months, dorsal MPFC might be implicated in triadic interactions, in which infants establish eye contact with others, in order direct their attention to specific objects/events in the external environment (see also Grossmann and Johnson, 2010). It should be stressed, however, that this segregation between dorsal and ventral regions is at odds with other studies from our Research Topic: on the one side, Farrow et al. implicate the dorsal (but not ventral) MPFC in the processing and evaluation of threatening words, picture and sounds; on the other hand, ventral (but not dorsal) MPFC is associated with processes related to social appraisal, such as the differential treatment of human and computer opponents in monetary transactions (Moretto et al.), or the conformity to the decision of in-group peers in a perceptual estimation task (Stallen et al.).

## Conclusions

In the last decades, psychologist and neuroscientists invested a considerable amount of research to investigate the ability to act “socially,” which is considered an evolutionary advantage of many species (Matusall). The present Research Topic is a collection of a large number (38) of original contributions from an interdisciplinary community which together highlight that determinants of individual social behavior should be best understood along at least two different dimensions. This general perspective represents the backbone for a comprehensive and articulated model of how people and their brains interact with each other in social contexts. However, despite its appeal, it remains unclear how the model put forward in this editorial relates to particular paradigms with high ecological value, where it is more difficult to neatly disentangle the relative contribution of personal/environmental or stable/transient determinants. This is for instance the case of Preston et al. who investigated hospitalized terminal patients, measuring the emotional reactions elicited in observers and whether they were related to the frequency with which aid was delivered. In this perspective, a great challenge for future research in social psychology and neuroscience will indeed be to develop more accurate predictive models of social behavior and to make them applicable to ecologically valid settings.

## Author contributions

All authors listed, have made substantial, direct and intellectual contribution to the work, and approved it for publication.

### Conflict of interest statement

The authors declare that the research was conducted in the absence of any commercial or financial relationships that could be construed as a potential conflict of interest.
